# Brain Microvascular Pericytes in Vascular Cognitive Impairment and Dementia

**DOI:** 10.3389/fnagi.2020.00080

**Published:** 2020-04-14

**Authors:** Maiko T. Uemura, Takakuni Maki, Masafumi Ihara, Virginia M. Y. Lee, John Q. Trojanowski

**Affiliations:** ^1^Institute on Aging and Center for Neurodegenerative Disease Research, Department of Pathology and Laboratory Medicine, Perelman School of Medicine, University of Pennsylvania, Philadelphia, PA, United States; ^2^JSPS Overseas Research Fellowship Program, Japan Society for the Promotion of Science, Tokyo, Japan; ^3^Department of Neurology, Kyoto University Graduate School of Medicine, Kyoto, Japan; ^4^Department of Neurology, National Cerebral and Cardiovascular Center, Osaka, Japan

**Keywords:** pericytes, mural cells, small vessel disease, vascular cognitive impairment and dementia, Alzheimer’s disease (AD), stroke, neurovascular coupling (NVC), blood–brain barrier (BBB)

## Abstract

Pericytes are unique, multi-functional mural cells localized at the abluminal side of the perivascular space in microvessels. Originally discovered in 19th century, pericytes had drawn less attention until decades ago mainly due to lack of specific markers. Recently, however, a growing body of evidence has revealed that pericytes play various important roles: development and maintenance of blood–brain barrier (BBB), regulation of the neurovascular system (e.g., vascular stability, vessel formation, cerebral blood flow, etc.), trafficking of inflammatory cells, clearance of toxic waste products from the brain, and acquisition of stem cell-like properties. In the neurovascular unit, pericytes perform these functions through coordinated crosstalk with neighboring cells including endothelial, glial, and neuronal cells. Dysfunction of pericytes contribute to a wide variety of diseases that lead to cognitive impairments such as cerebral small vessel disease (SVD), acute stroke, Alzheimer’s disease (AD), and other neurological disorders. For instance, in SVDs, pericyte degeneration leads to microvessel instability and demyelination while in stroke, pericyte constriction after ischemia causes a no-reflow phenomenon in brain capillaries. In AD, which shares some common risk factors with vascular dementia, reduction in pericyte coverage and subsequent microvascular impairments are observed in association with white matter attenuation and contribute to impaired cognition. Pericyte loss causes BBB-breakdown, which stagnates amyloid β clearance and the leakage of neurotoxic molecules into the brain parenchyma. In this review, we first summarize the characteristics of brain microvessel pericytes, and their roles in the central nervous system. Then, we focus on how dysfunctional pericytes contribute to the pathogenesis of vascular cognitive impairment including cerebral ‘small vessel’ and ‘large vessel’ diseases, as well as AD. Finally, we discuss therapeutic implications for these disorders by targeting pericytes.

## Introduction

**FIGURE 1:**
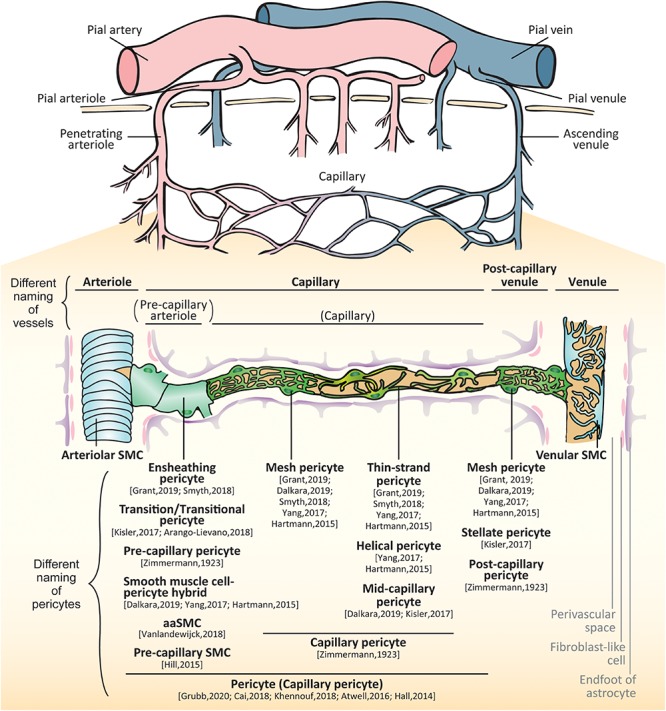
Brain vessels and mural cells. The pial arterioles branch from pial arteries which follow the outer rim of the brain via the meninges. The arterioles penetrate perpendicularly into the brain parenchyma (penetrating arteries) and further split into smaller arterioles. As their diameters and constituent cell types are changed, the vessels make a transition to capillaries. The capillary join to form venules that collect into pial venules and further into pial veins. In the small vessels, there are two types of mural cells separately located outside of endothelial layer: vascular smooth muscle cells (SMCs) and pericytes. SMCs are localized at the arteries, arterioles, venules and veins whereas pericytes are localized at the capillaries and post-capillary venules. The proximal branches coming off penetrating arterioles are sometimes called as pre-capillary arterioles. The subtypes of pericytes are differently called: ensheathing pericytes, transitional pericytes, pre-capillary pericytes, smooth muscle cell-pericyte hybrids, arteriole SMC (aaSMCs), or pre-capillary SMCs in a few branches from arterioles; capillary pericytes, mesh pericytes, thin-strand pericytes, helical pericytes, or mid-capillary pericytes in the middle part of capillary; mesh pericytes, stellate/stellate-like pericytes, or post-capillary pericytes in the post-capillary venules.

**FIGURE 2:**
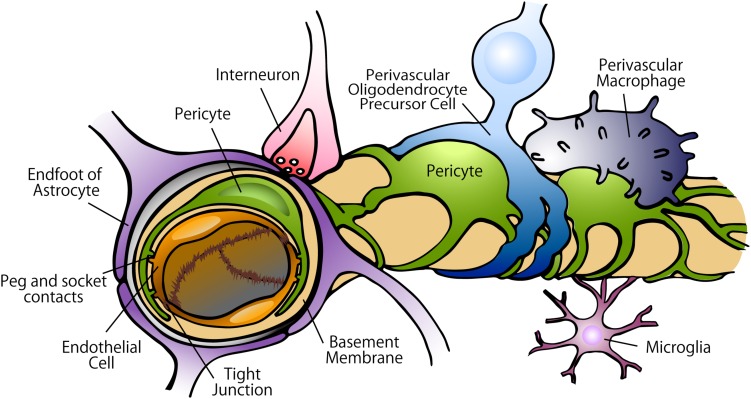
Constituents of the BBB in the capillary. In the BBB, tight junctions created by endothelial cells strictly regulate the movement of ions, molecules, and cells between the blood and the brain. The tight junctions are controlled by the cells surrounding the endothelium, including pericytes, astrocytes, perivascular OPCs, interneurons, perivascular macrophages, and microglia. Pericytes are localized on the abluminal surface of the endothelial layers and embedded in the basement membrane. Astrocytes extend polarized cellular processes that almost completely ensheath the vessel tubes.

Pericytes are mural cells, embedded within the basement membrane, and surrounding microvessels as illustrated in Figures 1 and 2. These cells were originally described in late 19th century (Eberth, 1871; Rouget, 1873) and initially named “pericytes” in 1923 by Zimmermann (Zimmermann, 1923) in accordance with their location enveloping the endothelium, and their being embedded in the basement membrane outside the microvessels (Zimmermann, 1923; Armulik et al., 2011; Geranmayeh et al., 2019). Although pericytes were considered to contribute to architectural maintenance and contraction of capillaries (Sandison, 1931; Zweifach, 1934; Clark and Clart, 1940), little had been known about their multifunctional characteristics and roles in neurological disorders until late 20th century (Brown et al., 2019). In the last 20 years, however, using a combination of markers and advancing technologies, a variety of functions of pericytes in health and disease have been revealed. Especially, microvascular pericytes in the central nervous system (CNS) have come into focus as they contribute to the maintenance of blood–brain barrier (BBB) (Armulik et al., 2010; Bell et al., 2010; Daneman et al., 2010; Quaegebeur et al., 2010), regulation of cerebral blood flow (CBF) (Peppiatt et al., 2006), and clearance of toxic waste products from the brain (Lendahl et al., 2019) as well as other multifunctional properties.

## Mural Cells in the Brain Small Vessels: Vascular Smooth Muscle Cells and Pericytes

The brain constitutes ∼2% of the adult human body weight but receives ∼20% of the cardiac output through CNS vascular network (Xing et al., 2017). In the brain, small vessels can be largely classified as three different types by their size and constituent cell types: (1) arterioles, (2) capillaries, and (3) venules (Attwell et al., 2016). There are gradual transitions between these vessel types; the transitions between arterioles and capillaries are called as pre-capillary arterioles while those between capillaries and venules are called as post-capillary venules (Dalkara and Alarcon-Martinez, 2015).

The arterioles branch from large arteries and follow the outer rim of the brain via the meninges (Bevan et al., 1999; Onodera, 2011). They penetrate perpendicularly into the cortex (penetrating arterioles), and upon entering the white matter, they begin to coil, loop, and spiral (Nonaka et al., 2003). Running through the brain parenchyma, the arterioles further split into smaller arterioles (Yamazaki and Kanekiyo, 2017). As their diameters and constituent cell types are changed, the vessels make a transition to capillaries. The capillaries then increase their diameter again and transition into the post-capillary venules, which join to form collecting venules that collect into larger veins (Landau and Davis, 1957; Harnarine-Singh et al., 1972; Onodera, 2011; Itoh and Suzuki, 2012; El-Bouri and Payne, 2016). In the small vessels (from the arterioles to venules), there are two types of mural cells separately located outside of endothelial layer: (1) vascular smooth muscle cells (SMCs) and (2) pericytes (Figure 1).

Smooth muscle cells and pericytes express shared mural cell markers including neuron-glial antigen 2 (NG2, or transmembrane chondroitin sulfate proteoglycan; CSPG4) platelet derived growth factor receptor beta (PDGFRβ), alanyl aminopeptidase (ANPEP, or CD13), vimentin, regulator of G protein signaling 5 (RGS5) (Itoh and Suzuki, 2012; Yang et al., 2017; Smyth L. et al., 2018; Vanlandewijck et al., 2018). Other mural cell markers such as α-smooth muscle actin (αSMA, or actin alpha 2, smooth muscle; ACTA2), transgelin (or smooth muscle protein 22-α; SM22α), calponin1 (CNN1), desmin, and melanoma cell adhesion molecule (MCAM, or CD146) are expressed more in SMCs than pericytes (Smyth L. et al., 2018; Zeisel et al., 2018). On the other hand, pericytes, but not SMCs, express ATP binding cassette subfamily C member 9 (ABCC9) (Bondjers et al., 2006) and preferentially internalize or take up the fluoroNissl dye NeuroTrace 500/525 when applied to the brain surface (Damisah et al., 2017). Most of the gene expression of pericytes, however, overlaps between SMCs and a certain subtype of pericytes. Furthermore, the expression of all these markers changes during growth and development, and may be up- or down-regulated in pathological conditions (Hughes and Chan-Ling, 2004; Armulik et al., 2011). Therefore, cell morphology and anatomical position should be taken into consideration to distinguish SMCs and pericytes. Table 1 provides anatomical differences in the cerebral small vessels and mural cell markers.

**TABLE 1:** Anatomical differences in brain small vessels and mural cell markers.

	**Arteriole**	**Proximal capillary**	**Mid-capillary**		**Post-capillary venule**	**Venule**	**References**
**Perivascular space**	^+ a^/+ (BG/WM)^b^/− (Cox)^c^	−	-		+	+	Zhang et al. (1990)^b^, Pollock et al. (1997)^c^, Salzman et al. (2005)^c^, Onodera (2011)^a^, Morris et al. (2016)^b^
**Mural cell**	SMC	Pericyte	Pericyte		Pericyte	SMC	Zimmermann (1923), Hall et al. (2014), Khennouf et al. (2018)
**Different naming of mural cell**		Ensheathing pericyte	Mesh pericyte	Thin-strand pericyte	Mesh pericyte	Stellate SMC	Dalkara (2019), Grant et al. (2019), Smyth L. et al. (2018), Yang et al. (2017)
		Transitional pericyte	Mid-capillary pericyte		Stellate pericytes		Kisler et al. (2017a), Arango-Lievano et al. (2018), Dalkara (2019)
		Pre-capillary pericyte	Capillary pericyte		Post-capillary pericyte		Zimmermann (1923)
		Smooth muscle-pericyte hybrid					Hartmann et al. (2015), Yang et al. (2017), Dalkara (2019)
		aaSMC					Vanlandewijck et al. (2018)
		Pre-capillary SMC					Hill et al. (2015)
**SMCs and pericytes**							
CSPG4 (NG2)	+	+ ⁣ +	+ ⁣ +		+ ⁣ +	+	Hartmann et al. (2015), Hill et al. (2015), Yang et al. (2017), Smyth L.C.D. et al. (2018), Vanlandewijck et al. (2018)
PDGFRβ	+	+ ⁣ +	+ ⁣ +		+ ⁣ +	+	Hartmann et al. (2015), Yang et al. (2017), Smyth L.C.D. et al. (2018), Vanlandewijck et al. (2018)
ANPEP (CD13)	+	+	+		+	+	Kunz et al. (1994), Yang et al. (2017), Smyth L.C.D. et al. (2018)
Vimentin	+ ⁣ +	+	+		+	+ ⁣ +	Nehls and Drenckhahn (1993), Itoh and Suzuki (2012)
RGS5	+	+	+		+	+	Bondjers et al. (2006), Özen et al. (2014), Yang et al. (2017)
**SMCs preferential**							
ACTA2 (αSMA)	+ ⁣ + ⁣ +	+	±		±	+ ⁣ +	Boado and Pardridge (1994), Bandopadhyay et al. (2001), Itoh and Suzuki (2012), Damisah et al. (2017), Yang et al. (2017), Alarcon-Martinez et al. (2018), Smyth L.C.D. et al. (2018)
Transgelin	+ ⁣ +	+	−		−	±	Smyth L.C.D. et al. (2018),
CNN1	+ ⁣ +	±	−		−	−	Berthiaume et al. (2018), Vanlandewijck et al. (2018)
Desmin	+ ⁣ +	− or ±	− or ±		− or ±	+ ⁣ +	Nehls and Drenckhahn (1991), Itoh and Suzuki (2012), Smyth L.C.D. et al. (2018)
MCAM (CD146)	+ ⁣ +	±	±		±	+ ⁣ +	Smyth L.C.D. et al. (2018)
**Pericytes preferential**							
ABCC9	−	±	+ ⁣ +		+ ⁣ +	+	Bondjers et al. (2006), Berthiaume et al. (2018), Vanlandewijck et al. (2018)
Fluoro-Nissl dye	−	+	+ ⁣ +		+ ⁣ +	±	Damisah et al. (2017)

*ABCC9, ATP binding cassette subfamily C member 9; ACTA2, actin alpha 2, smooth muscle; ANPEP, alanyl aminopeptidase, membrane; BG, basal ganglia; CNN1, calponin 1; Cox, cerebral cortex; CSPG4, chondroitin sulfate proteoglycan 4; MCAM, melanoma cell adhesion molecule; PDGFRβ, platelet derived growth factor receptor beta; RGS5, regulator of G protein signaling 5; WM, white matter.*

The classification of small vessels is sometimes complicated and controversial because of the definition of constituent mural cells. Although there is a consensus that SMCs are located in arterioles and venules as well as larger arteries and veins (Iadecola, 2017; Sweeney et al., 2018), the classification and nomenclature of pericyte-surrounding vessels have been greatly debated mainly due to the heterogeneity of pericytes (Cheng et al., 2018). Pericytes in the capillaries gradually transition to SMCs in the arterioles; drawing a clear line between those vessels is quite difficult (Zimmermann, 1923). Originally, Zimmermann defined pericytes including their transition form to SMCs, residing on the three consecutive vessels, namely, (1) pre-capillary arterioles, (2) capillaries, and (3) post-capillary venules (Zimmermann, 1923). Zimmermann therefore differently named the pericytes on each vessel: (1) pre-capillary pericytes, at the last arterial ends that merge into the capillary system; (2) capillary pericytes, at the capillaries in the narrowest sense; and (3) post-capillary pericytes, on post-capillary venules up to veins showing regular, fusiform smooth muscle fibers.

As techniques such as three-dimensional live imaging have been developed, the branching order coming off penetrating arterioles has also been taken into consideration to define the vessels in rodent brains. The definition of the vessels, however, has varied depending on the studies. While some studies have defined all vessels including proximal and distal branches coming off penetrating arterioles as capillaries (Peppiatt et al., 2006; Hall et al., 2014; Cai et al., 2018; Khennouf et al., 2018; Grubb et al., 2020), others have defined proximal branches as pre-capillary arterioles (Fernández-Klett et al., 2010; Hartmann et al., 2015; Hill et al., 2015). To make matters more complicated, Hill et al. (2015), have asserted that the mural cells on the proximal branches coming off arterioles should be called as SMCs, which have provided confusion in the field with the result that different members of the field use different terminologies and definitions about pericytes and pericyte-residing vessels (Hartmann et al., 2015; He L. et al., 2016; Kisler et al., 2017a; Yang et al., 2017; Smyth L. et al., 2018; Dalkara, 2019; Grant et al., 2019). Nowadays, to avoid confusion, the researchers have claimed that the capillaries should include transition to the arterioles, and the mural cells on those capillaries should be called as “pericytes” (Attwell et al., 2016). In this review, we will describe the differences of mural cells in the small vessels, namely, (1) SMCs on the arterioles and venules and (2) pericytes on the capillaries and post-capillary venules, introducing the different terminology of pericytes. Thereafter, we will focus on the pericyte function and dysfunction in health and diseases.

### Arteriolar SMCs

In the arterioles, SMCs continuously enwrap the abluminal side of endothelial cell layer and make myoendothelial gap junction (Aydin et al., 1991). The SMCs in the arterioles have an inconspicuous soma and extend broad processes (Attwell et al., 2016) that strongly express αSMA transgelin, desmin, CD146, and CNN1 as well as shared mural cell markers including NG2, PDGFRβ, CD13, vimentin, and RGS5. Outside of the SMCs is perivascular space with fibroblast-like cells, surrounded by collagen layer and endfeet of astrocytes (Mastorakos and McGavern, 2019).

### Capillary Pericytes

In the capillaries, the vessel size further decreases, and the endothelial layer is intermittently surrounded by pericytes. Compared to the peripheral vascular beds, CNS capillaries have higher pericyte-to-endothelial cell ratios (1:1 to 1:3) and around 70–80% of the capillary surface area is covered with pericyte cell processes (Bell et al., 2010; Winkler et al., 2010, 2014). Capillary pericytes have a conspicuous protruding ovoid cell body with long thin processes that course along the capillary for longer distances and are embedded within the basement membrane.

The role of capillary pericytes in CBF control has long been debated. Using mouse models, some researchers have reported that arteriolar SMCs but not capillary pericytes regulate CBF in response to neuronal activities or ischemic stress (Fernández-Klett et al., 2010; Hill et al., 2015). Others, however, have shown that capillary pericytes also change the vessel diameter and CBF by stimuli, neuronal activation, or ischemia (Peppiatt et al., 2006; Yemisci et al., 2009; Hall et al., 2014; Pieper et al., 2014; Kisler et al., 2017b; Rungta et al., 2018), suggesting capillary pericytes also contribute to CBF regulation.

Because pericytes are morphologically and functionally heterogeneous, pericytes are sometimes subclassified according to their topology, morphology, and the protein expression levels.

#### Pericytes in the Proximal Capillaries

The studies of mouse brain cortices using two-photon microscopy have revealed the morphological and functional distinction of the pericytes on the proximal branches (mostly up to 2nd or 4th order) coming off penetrating arterioles from those on the higher branch-order capillaries or larger arterioles (Hartmann et al., 2015; Hill et al., 2015; Kisler et al., 2017a; Yang et al., 2017; Smyth L. et al., 2018; Grant et al., 2019). The pericytes located in this point of transition possess highly visible and protruding ovoid soma with thin and circumferential processes enveloping the vessels. These cells express more αSMA than pericytes in mid-capillaries, but not as much as SMCs in the penetrating arterioles (Alarcon-Martinez et al., 2018; Grant et al., 2019). Aside from αSMA, the cells also express desmin and transgelin, but hardly express CNN1 (Smyth L. et al., 2018; Vanlandewijck et al., 2018).

Because these pericytes are positioned at the transition between arterioles and capillaries, and have shared some characteristics with SMCs, the terminology and classification of these mural cells have been hotly debated. The cells have been variably called as ensheathing pericytes (Smyth L. et al., 2018; Grant et al., 2019), transition/transitional pericytes (Kisler et al., 2017a; Arango-Lievano et al., 2018), pre-capillary pericytes (Zimmermann, 1923), simply ‘pericytes’ or ‘capillary pericytes’ (Hall et al., 2014; Attwell et al., 2016; Cai et al., 2018; Khennouf et al., 2018; Grubb et al., 2020), smooth muscle-pericyte hybrids (Hartmann et al., 2015; Yang et al., 2017; Dalkara, 2019), arteriole SMC (aaSMC) (Vanlandewijck et al., 2018), or pre-capillary SMCs (Hill et al., 2015).

At the same time, the vessels of proximal branches coming off penetrating arterioles are also differently defined as pre-capillary arterioles (Fernández-Klett et al., 2010; Hartmann et al., 2015; Hill et al., 2015; Berthiaume et al., 2018; Erdener and Dalkara, 2019; Grant et al., 2019), post-arteriole capillaries (Gould et al., 2016) or a part of capillaries (Hall et al., 2014; Attwell et al., 2016; Cai et al., 2018; Khennouf et al., 2018; Grubb et al., 2020).

The cells in this proximal branches (especially, 1st to 2nd branches) coming off arterioles has drawn attention as they highly contribute to neurovascular coupling (NVC) (Hall et al., 2014; Hill et al., 2015; Cai et al., 2018; Khennouf et al., 2018; Rungta et al., 2018; Grubb et al., 2020) and no-reflow phenomenon after acute ischemia (Hall et al., 2014; Hill et al., 2015). Although referring to these mural cells as a subtype of “pericytes” has become almost a consensus, the shifting nomenclature of these cells and vessels has been the root of recent controversies on pericyte roles as regulators of CBF (discussed below).

#### Pericytes in the Mid-Capillaries

In the mouse brain, pericytes in the mid-capillaries are divided into two subtypes according to the morphology of their processes, namely, (1) mesh pericyte and (2) thin-strand pericyte or helical pericyte (Hartmann et al., 2015; Yang et al., 2017; Smyth L. et al., 2018; Dalkara, 2019; Grant et al., 2019). The mesh pericytes adopt a mesh-like appearance and are located on the proximal side of a capillary with higher coverage area than thin-strand pericytes (Hartmann et al., 2015; Grant et al., 2019). The thin-strand pericytes or helical pericytes extend thin, meandering processes that run along the vessel lumen. These two types of pericytes express NG2, CD13, and PDGFRβ, and slightly express CD146 and αSMA, but hardly express desmin, transgelin, nor CNN1 (Alarcon-Martinez et al., 2018; Smyth L. et al., 2018; Vanlandewijck et al., 2018; Zeisel et al., 2018). Instead, pericytes express ABCC9 (Bondjers et al., 2006; Vanlandewijck et al., 2018) and preferentially take up the fluoroNissl dye NeuroTrace 500/525 (Damisah et al., 2017). The pericytes in these capillaries play vital roles for BBB maintenance and small molecule transport (Armulik et al., 2010; Bell et al., 2010; Liu et al., 2012).

### Post-capillary Pericytes

In the post-capillary venules, different shaped mesh pericytes, also called stellate/stellate-shaped pericytes, surround the endothelial layer. These cells have many slender and shorter branching processes than capillary pericytes (Hashitani and Lang, 2016; Yang et al., 2017; Arango-Lievano et al., 2018). They express αSMA, ABCC9, cysteine sulfinic acid decarboxylase (P-selectin), and endomucin (Marín-Padilla, 2012). The expression level of αSMA is lower than the mural cells in the arterioles and proximal capillaries (Grant et al., 2019). The pericytes in the post-capillary venules are thought to regulate immune cell entry to the brain parenchyma like those in other tissues (Proebstl et al., 2012; Stark et al., 2013; Attwell et al., 2016; Dalkara, 2019; Rudziak et al., 2019). Outside of the pericytes are astroglial end-feet forming glia limitans. Between the endothelial basement membrane and astrocytic basement membrane is perivascular space, where antigen presenting cells reside (Engelhardt et al., 2017; Mastorakos and McGavern, 2019). Fibroblast-like cells are within the astrocytic basement membrane (Mastorakos and McGavern, 2019).

### Venular SMCs

Post-capillary venules are collected to form ascending venules. In the venules, the endothelial cell layer is surrounded by stellate-shaped SMCs with broad leaf-like processes (Ushiwata and Ushiki, 1990; Armulik et al., 2011). Venous SMCs express NG2, CD13, PDGFRβ, αSMA, transgelin, ABCC9, but not CNN1 (Vanlandewijck et al., 2018). And the expression level of αSMA and transgelin in the venous SMCs is lower than the mural cells in the arterioles (Vanlandewijck et al., 2018; Grant et al., 2019). SMCs in brain venules express NG2, which is different from the venules of peripheral tissues (Murfee et al., 2005; Stark et al., 2013). Outside of SMCs are perivascular space and fibroblast-like cells (Mastorakos and McGavern, 2019).

## Functions of Pericytes

### BBB Maintenance, Angiogenesis, and Vessel Stabilizing

The CNS vascular system possess a highly selective semipermeable border formed by the BBB wherein tight junctions and adherens junctions created by endothelial cells strictly regulate the movement of ions, molecules, and circulating cells between the blood and the brain (Luissint et al., 2012; Daneman and Prat, 2015). The tight and adherens junctions are controlled by various types of cells surrounding the endothelium, such as pericytes, astrocytes, perivascular oligodendrocyte precursor cells (OPCs), interneurons, perivascular macrophages, microglia, and other immune cells (Abbott et al., 2010; Seo et al., 2014; Faraco et al., 2017; Stebbins et al., 2019) (Figure 2).

Capillary pericytes play especially crucial roles in the function of the BBB. Pericyte ablation leads to breakdown of the BBB in the mouse brain (Nikolakopoulou et al., 2019). Pericytes control protein expression in the tight junctions, their alignment with endothelial cells, and the bulk-flow transcytosis of fluid-filled vesicles across the BBB (Armulik et al., 2010; Bell et al., 2010; Daneman et al., 2010; Quaegebeur et al., 2010).

Pericytes also play a key role in the generation of new blood vessels. During angiogenesis, a complex web of bidirectional signaling pathways between endothelial cells and pericytes is essential for forming and stabilizing new blood vessels (Gaengel et al., 2009; Stapor et al., 2014). The signaling molecules involved in these processes include platelet-derived growth factor B (PDGFB)/PDGF receptor beta (PDGFRβ), transforming growth factor beta (TGFβ), Notch, vascular endothelial growth factor (VEGF), sphingosine-1 phosphate (S1P)/S1P receptor 1 (S1PR1 or EDG), and angiopoietin 1 and 2 (ANGPT1, ANGPT2)/TEK receptor tyrosine kinase (TEK, or TIE2) all of which differentially contribute to these signaling activities (Liu et al., 2000; Winkler et al., 2011b; Zechariah et al., 2013; Eilken et al., 2017; Teichert et al., 2017; Cheng et al., 2018). During angiogenesis, pericytes are reported to be recruited from the bone marrow as well as brain parenchyma in response to the PDGF-BB secreted from endothelial cells (Rajantie et al., 2004; Song et al., 2005; Kokovay et al., 2006; Gaengel et al., 2009). On the other hand, pericytes induce endothelial cell sprouting and stabilization via secreting TGFβ, VEGF, and ANGPT1 (Paik et al., 2004; Durham et al., 2014; Teichert et al., 2017; Blocki et al., 2018). Lack of pericytes leads to endothelial hyperplasia and abnormal vascular morphogenesis including microaneurysm (Lindahl et al., 1997; Hellström et al., 2001). When a single brain pericyte is ablated, the processes from neighboring pericytes are extended to contact uncovered regions of the endothelial cells and maintain the vessel diameter and vessel stability (Berthiaume et al., 2018).

### Regulation of Capillary Diameter and Blood Flow

Cerebral blood flow is dynamically altered in response to changes of transient neuronal activity, which is referred to as NVC (Abbott et al., 2010; Attwell et al., 2010; Kisler et al., 2017a). It is controlled by the cells within the neurovascular unit (NVU) including endothelial cells, pericytes, SMCs, astrocytes, OPCs, and neurons (Peppiatt et al., 2006; Attwell et al., 2010; Hall et al., 2014; Mishra et al., 2016; Kisler et al., 2017a, b; Rungta et al., 2018). In response to the different neurotransmitters, pericytes dilate capillaries and increase local CBF (Hamilton et al., 2010). In pathological conditions such as ischemic stroke (Hall et al., 2014; Yemisci et al., 2009) and AD (Nortley et al., 2019), brain capillaries are constricted by pericytes. In ischemic stroke mouse brains, damaged and dead pericytes squeeze the capillaries and sustain the reduction of CBF even after recanalization of the larger vessels, causing the no-reflow phenomenon (Yemisci et al., 2009; Kloner et al., 2018). The burden of amyloid beta (Aβ) oligomer causes pericyte contraction and capillary stenosis, which decreases CBF in the AD brains (Nortley et al., 2019).

The role of pericytes in regulation of vessel diameter has been heatedly debated. When Rouget first describe the branched cells on the capillary wall, which is nowadays called as pericytes, he regarded them as contractile cells (Rouget, 1873). Thereafter, the studies which supported or objected to the pericyte contractility were successively reported (Krueger and Bechmann, 2010). In terms of CBF regulation, SMCs located at arterioles were traditionally thought to control CBF (Iadecola, 2004). This view of CBF dynamics was revolutionized by the findings that capillary diameter also changes with neural activity (Peppiatt et al., 2006; Hall et al., 2014; Kisler et al., 2017b; Khennouf et al., 2018; Rungta et al., 2018) and ischemia (Yemisci et al., 2009; Hall et al., 2014). Furthermore, the loss of pericytes has been reported to lead to diminishing CBF in response to functional hyperemia in pericytes-deficient mice (Bell et al., 2010; Kisler et al., 2017b, 2020). However, Hill et al. (2015) refuted that pericytes are involved in the regulation of CBF, and put forward the view that arteriolar SMCs may be the key players regulating CBF. Fernández-Klett et al. (2010) also showed pre-capillary and penetrating arterioles, but not pericyte in capillaries are responsible for the CBF increase induced by neural activity. These controversial reports most likely stem from the different definitions of pericytes in the proximal capillaries. Some of these reports concur that the mural cells in the proximal branches coming off penetrating arterioles respond to the stimulations outlined above and change vessel diameters accordingly (Fernández-Klett et al., 2010; Hall et al., 2014; Hill et al., 2015; Cai et al., 2018; Khennouf et al., 2018). Pericytes residing at the proximal capillaries possess both characteristics of pericytes and SMCs (Hartmann et al., 2015), which may lead to discrepant interpretations by different investigators. Some groups showed stimulation-evoked increases in synaptic activity and capillary dilation starting mostly at the first- or second-order capillary then propagating along arterioles and downstream capillaries (Cai et al., 2018; Khennouf et al., 2018), which may position the pericytes in the proximal capillaries as the major regulators of CBF. In addition, Grubb et al. (2020) reported that a pre-capillary sphincter, at the junction between the penetrating arteriole and first order branch, modulated capillary flow while protecting the downstream capillary bed from adverse pressure fluctuations. Taken together, the proximal branches coming off arterioles seem to be the gatekeeper that controls CBF in the capillary beds.

### Clearance of Materials From the Brain

Pericytes internalize small molecules and neurotoxic blood-derived products which enter the breached BBB (i.e., immunoglobulins, fibrin and albumin) through receptor-mediated endocytosis or non-specific pinocytosis (Armulik et al., 2010; Bell et al., 2010; Schultz et al., 2017). Pericytes also internalize large solid substance through phagocytosis. Engulfed molecules are transported to lysosomes for enzymatic degradation (Diaz-Flores et al., 2009) or possibly transported to the blood circulation (Zhao et al., 2015). While tumor necrosis factor alpha (TNFα) and interferon-γ (IFNγ) enhance phagocytic uptake, TGFB1 attenuates phagocytic uptake in pericytes.

Pericytes may clear substances derived from the brain parenchyma as well as around vessels. Pericyte loss aggravates Aβ deposition in transgenic mice (Sagare et al., 2013). Aβ clearance by pericytes is mainly performed through receptor-mediated endocytic pathways, especially low-density lipoprotein receptor-related protein 1 (LRP-1) (Shibata et al., 2000; Zlokovic et al., 2010; Ma et al., 2018).

Another clearance system which might be related to pericytes is the CNS lymphatic drainage system. In the CNS, there are two major extracellular fluids, namely, (1) cerebrospinal fluid (CSF) and (2) interstitial fluid (ISF). CSF drains to cervical lymph nodes via the cribriform plate and nasal lymphatics (Kida et al., 1993; Spector et al., 2015), as well as via dural lymphatics (Aspelund et al., 2015; Louveau et al., 2015; Absinta et al., 2017; Ahn et al., 2019) and along cranial nerves (Hatterer et al., 2006; Aspelund et al., 2015). ISF containing metabolic products of the brain as well as Aβ and tau drains to lymph nodes by the shared or distinct pathways from CSF (Engelhardt et al., 2017; Cheng and Wang, 2020). Two different pathways for draining ISF to the periphery — the perivascular and paravascular pathways — are controversially proposed (Engelhardt et al., 2016). In the perivascular pathway, ISF and solutes from CNS parenchyma enter the basement membranes of capillaries, where pericytes are embedded, and drain directly via tunica media of arterioles and arteries out of the brain to cervical lymph nodes (Carare et al., 2008, 2014; Engelhardt et al., 2016). Paravascular pathway, also known as glymphatic system, denotes the moving of CSF into the brain along arterial perivascular spaces and successively into the interstitium to mix with ISF, which then guides flow toward the venous perivascular spaces, removing metabolic waste of ISF to the CSF via convective bulk flow (Iliff et al., 2012). In the glymphatic system, astrocytes are important for ion buffering and fluid exchange between the CSF and ISF (Jessen et al., 2015). The exchange through the glymphatic system is suggested to be dependent on the water channel aquaporin-4 (AQP4) located in astrocytic endfeet (Iliff et al., 2012). However, it remains enigmatic whether or not AQP4 is solely responsible for this fluid transport or not (Lendahl et al., 2019). Recently, insufficient PDGFB signaling in the *Pdgfb*^*ret/ret*^ mice has shown decreased pericyte coverage of the vessels with decreased AQP4 polarization to astrocyte endfeet, which impairs maturation of the glymphatic function (Munk et al., 2019). The focal absence of pericytes correlates with relocation of AQP4 from astrocytic endfeet to the soma of astrocytes (Armulik et al., 2010). Pericytes express laminin-α2 (LAMA2), laminin-β1, and laminin-γ1, which encode the subunits of laminin 211 (Vanlandewijck et al., 2018). Laminin 211 deposits in the vascular basement membrane and interacts with dystrophin in astrocytes, which acts as a molecular bridge to AQP4 to keep it in the astrocyte endfeet (Guadagno and Moukhles, 2004). Indeed, *Lama2* knockout in mice results in BBB abnormalities in association with loss of AQP4 polarization to astrocyte endfeet (Menezes et al., 2014). The above referenced reports suggest that pericytes might influence the development of the glymphatic system through deposition of laminin 211 in the vascular basement membrane, which maintains the polarization of AQP4 at astrocytic endfeet. However, there are critical assessments of the proposed glymphatic system (Hladky and Barrand, 2014, 2019; Abbott et al., 2018). Several observations or simulations do not support the glymphatic mechanism (Jin et al., 2016; Smith et al., 2017) nor convective fluid flow of CSF (Asgari et al., 2016; Holter et al., 2017). Hence, the existence of the paravascular pathway as a CNS drainage system is still under debate.

### Inflammation and the Regulation of Immune Cells

Brain pericytes have many properties of immune regulating cells such as (1) responding to and expressing pro-inflammatory and anti-inflammatory molecules, (2) regulating leukocyte extravasation and trafficking, and (3) controlling immune cell activation including T cells, macrophages, and microglia (Rustenhoven et al., 2017; Thomas et al., 2017; Duan et al., 2018; Smyth L.C.D. et al., 2018). In the mouse brain, pericytes function as the initial sensor of systemic inflammation and relay the infection signal to neurons by secreting chemokine CC chemokine ligand 2 (CCL2, also known as monocyte chemotactic protein-1, MCP1) (Duan et al., 2018).

Pericytes express and release several mediator molecules that enhance leukocyte extravasation. Although the endothelial cells are well known to induce leukocyte crawling and extravasation (Muller, 2002), pericytes also contribute to leukocyte transmigration (Proebstl et al., 2012). *In vivo* observation of mouse skin vessels have demonstrated that leukocyte extravasation occur only post-capillary venular pericytes (Stark et al., 2013). After inflammation stimuli, neutrophils exhibited transendothelial migration (TEM) and sub-endothelial cell crawling along pericyte processes, which was supported by pericyte-derived intercellular adhesion molecule-1 (ICAM-1) and its leukocyte integrin ligands, macrophage-1 antigen (Mac-1) and lymphocyte function–associated antigen-1 (LFA-1). Then, the leukocytes transmigrated to the interstitium through the gaps between adjacent pericytes (Proebstl et al., 2012). After extravasation, the leukocytes interact with capillary pericytes as well. Pericyte-monocyte interaction is mediated mainly by macrophage migration-inhibitory factor (MIF) and CCL2, whereas neutrophil migration involves MIF and C-X3-C motif chemokine ligand 1 (CXCL8, also known as interleukin 8, IL8) (Stark et al., 2013).

Exposure of pericytes to cytokines such as interleukin 1 beta (IL1β) and TNFα triggers the release of inflammatory molecules and matrix metalloprotease 9 (MMP9), leading to BBB breakdown *in vitro* (Herland et al., 2016). The immunomodulatory factors secreted by pericytes including IL1β, TNFα, IFNγ, and interleukin 6 (IL6) induce a proinflammatory state in astrocytes, microglia, and endothelial cells, and cause apoptotic neuronal death (Kovac et al., 2011; Matsumoto et al., 2018).

Conversely, pericytes can also secrete several anti-inflammatory substances such as interleukin 33 (IL33) and C-X3-C motif chemokine ligand 1 (CX3CL1) (Rustenhoven et al., 2016, 2017; Yang et al., 2016), both of which are shown to promote anti-inflammatory microglial phenotype in mouse models (Cardona et al., 2006; Fu et al., 2016). Furthermore, depletion of pericytes induced inflammatory responses in endothelial cells and perivascular infiltration of macrophages in mouse retinal vessels, suggesting pericytes exerts an anti-inflammatory effect on endothelial cells under normal conditions (Ogura et al., 2017).

### Phenotype Changes

Pericytes display some similarities to mesenchymal stem cells (Wong et al., 2015). Responding to the stimuli and environmental changes, pericytes may transform into multipotent stem cells and differentiate into various cells including neural, vascular, and glial cells (Dore-Duffy et al., 2006; Nakagomi et al., 2015b; Pombero et al., 2016). Pericytes extracted from ischemic mouse brain and human brain pericytes under oxygen-glucose deprivation states develop stem properties *in vitro* (Nakagomi et al., 2015a). Pericytes under ischemic condition *in vivo* and *in vitro* are also reported to acquire a microglial phenotype corresponding with increased phagocytic property (Özen et al., 2014; Sakuma et al., 2016).

These phenotype changes of pericytes under stimulation can be beneficial for the compensatory remodeling after brain injury and ischemia, rapid response to infection and inflammation, and clearing compromised cells or neurotoxic substances breaching an impaired BBB. However, no multipotency of pericytes in aging and injury *in vivo* has been reported, challenging the current view of pericytes as tissue-resident multipotent progenitors (Guimaraes-Camboa et al., 2017).

### Scar Formation

Central nervous system injury evokes the recruitment of astroglia and scar formation. Pericytes and OPCs as well as astrocytes are observed within glial scars. After spinal injury or ischemic stroke, pericytes proliferate and migrate to the injured region and form a glial scar (Göritz et al., 2011; Makihara et al., 2015; Dias et al., 2018; Hesp et al., 2018). Extracellular matrix proteins, such as periostin have shown to be expressed in the extracellular space of the injury region, which induces pericyte proliferation and leads to scar formation (Yokota et al., 2017). The glial scar around the injury site forms a barrier between the injured and the non-injured tissue to prevent further neuronal loss, which eventually hinders the axonal regeneration in the scarred area (Zhu et al., 2015; Anderson et al., 2016; Cheng et al., 2018; Dias et al., 2018). Recent evidence has demonstrated that the glial scar can also promote CNS regeneration after injury (Anderson et al., 2016), suggesting a dual function. The complexity and heterogeneity of the glial scar derived from different cell types (i.e., astrocytes, pericytes, and OPCs) at various phases in CNS diseases remains to be elucidated.

## Cross Talk of Pericytes With Vascular Cells and Glia

### Endothelial Cells and Pericytes

Pericytes and endothelial cells are connected to a shared basement membrane by several types of integrin molecules. In areas lacking the basement membrane, interdigitations of pericytes and endothelial cell membranes, called peg and socket contacts, form direct connections by N-cadherin and connexin 43 (Armulik et al., 2005; Winkler et al., 2011b). The crosstalk between pericytes and endothelial cells is indispensable for angiogenesis, vascular stability, and BBB formation. For CNS pericytes and endothelial cells, PDGFB/PDGFRβ, TGFβ, Notch, VEGF, and S1P/S1PR1 signaling events are well investigated (Darland et al., 2003; Paik et al., 2004; Gaengel et al., 2009; Walshe et al., 2009; Liu et al., 2010; Li et al., 2011). ANGPT signaling is investigated in the retinal endothelial cells and pericytes (Winkler et al., 2011b).

In the angiogenesis of the mouse brain, PDGF-BB secreted by endothelial cells recruits PDGFRβ-positive pericytes and progenitor cells (Tallquist et al., 2003; Gaengel et al., 2009). PDGFB signaling also stimulates pericyte proliferation (Geranmayeh et al., 2019), and sustained PDGF-BB–PDGFRβ signaling in the adult CNS is required for pericyte cell survival (Geraldes et al., 2009; Bell et al., 2010).

TGFβ signaling is vital for microvessel stability affecting both endothelial cells and pericytes. Endothelially secreted TGFβ regulates differentiation of pericyte progenitors (Ribatti et al., 2011) and induces pericyte contractile protein expression and extracellular matrix production and facilitates proper pericyte attachment in coordination with Notch signaling (Li et al., 2011; Winkler et al., 2011a). Pericyte-derived TGFβ contributes to endothelial maturation through SMAD signaling (Winkler et al., 2011b).

Vascular endothelial growth factor produced by pericytes and endothelial cells also shows reciprocal interaction (Sweeney et al., 2016). Pericyte-derived VEGF in the mouse brain promotes endothelial sprouting and cell survival (Franco et al., 2011; Eilken et al., 2017). VEGF treatment enhances pericyte coverage of brain endothelial cells with increased N-cadherin production (Zechariah et al., 2013). VEGF induces proliferation and migration of pericytes as well as endothelial cell stabilization (Darland et al., 2003).

S1P is originally described as secreted by endothelial cells. Its receptor, S1PR1 is expressed in mural cells including pericytes. S1P secreted by endothelial cells is essential for pericytes coverage in the mouse brain (Allende et al., 2003) and stabilizes endothelial/pericyte cell adhesion through N-cadherin (Paik et al., 2004; Gaengel et al., 2009) and maintains the BBB (Yanagida et al., 2017). Human pericytes secrete S1P, which induces the expression of adhesion proteins in human retinal endothelial cells *in vitro* (McGuire et al., 2011).

ANGPT1 and ANGPT2 differently contribute to angiogenesis. The ANGPT1 is mainly expressed in pericytes and ANGPT2 is mainly expressed in endothelial cells. The ligand of ANGPT, TEK is mainly expressed in endothelial cells (Sundberg et al., 2002). Pericyte-derived ANGPT1 activates endothelial TEK and promotes endothelial survival (Geevarghese and Herman, 2014). TEK is also expressed at lower levels by pericytes and its downstream signaling in pericytes is essential for angiogenesis (Teichert et al., 2017). In angiogenesis, ANGPT2 was thought to antagonize ANGPT1, but later was found to act as both agonist/antagonist of TEK signaling in the endothelium (Yuan et al., 2009; Akwii et al., 2019). ANGPT2 expressed by mouse endothelial cells leads to the dissociation of TEK expressing pericytes from vessels, which initiates endothelial cell sprouting (Armulik et al., 2005).

Crosstalk between pericytes and endothelial cells is also mediated by circular RNA. Diabetes-related stress up-regulates a circular RNA, *cPWWP2A* (PWWP domain containing 2A) expression in pericytes, which inhibit *microRNA-579* and regulate vascular integrity (Liu C. et al., 2019).

### Astrocytes and Pericytes

The crosstalk between pericytes and astrocytes contributes to BBB maintenance, NVC, and white matter attenuation under chronic hypoperfusion (Bonkowski et al., 2011).

Pericytes facilitate the attachment of astrocyte endfeet to the BBB (Ihara and Yamamoto, 2016; Geranmayeh et al., 2019) and pericyte-deficient mice lose AQP4 in the endfeet of astrocytes (Armulik et al., 2010). On the other hand, astrocytes control pericyte migration, differentiation, and the juxtaposition of pericytes to endothelial cells (Nakagawa et al., 2009; Yao et al., 2014). Astrocyte-derived apolipoproteins differently regulate cyclophilin A (CypA) signaling in pericytes, which controls BBB integrity (Bell et al., 2012). In NVC, astrocytic calcium signaling mediates capillary dilation via pericytes (Mishra et al., 2016; Kisler et al., 2017a).

### Oligodendrocyte Precursor Cells and Pericytes

Oligodendrocyte precursor cells (OPCs) have recently emerged as one of the contributors to BBB. According to their regional differences, OPCs can be divided into two subtypes, namely, perivascular OPCs and parenchymal OPCs (Maki, 2017; Kishida et al., 2019). In the human and mouse brain, perivascular OPCs are attached to cerebral endothelial cells and pericytes through basal lamina, and thereby are thought to become novel components of the BBB (Seo et al., 2013; Maki et al., 2015). OPC-specific TGFβ1 depleted mice exhibited cerebral hemorrhage and loss of BBB function, showing the role of OPCs for BBB maintenance through TGFβ1 signaling (Seo et al., 2014). *In vitro* experiments have shown that OPC-derived factors increase pericyte proliferation whereas pericyte-derived factors support OPC self-renewal and differentiation (Maki et al., 2015, 2018). In the developing mouse forebrain, pericyte-derived TGFβ family proteins contribute to the migration and distribution of OPCs in brain parenchyma (Choe et al., 2014), while perivascular OPC migration to the vessels in the developing CNS requires interaction with endothelium but not pericytes (Tsai et al., 2016).

In the adult mouse brain, pericytes respond to toxin-induced demyelination in the brain and stimulate OPC differentiation during remyelination through Lama2 (De La Fuente et al., 2017). Pericyte-derived Lama2 also instructs neuronal stem cells to an oligodendrocyte fate (Silva et al., 2019).

A recent report has shown that odor triggers rapid Ca^2+^ elevations in OPC processes before pericytes and SMCs dilate the vessels responding to synaptic activation, suggesting possible relationship between OPCs and pericytes in the NVC (Rungta et al., 2018).

### Microglia and Pericytes

Microglia have been regarded as the main executor of inflammation after acute and chronic CNS disorders. The interaction between microglia and vascular cells – including pericytes – has important roles for vascular inflammation, angiogenesis, and BBB integrity (Ding et al., 2018; Thurgur and Pinteaux, 2019). Although endothelial cells are thought to be the main source of cytokines and chemokines which trigger microglial activation upon vascular inflammation, pericytes are also known to be key mediators in this process. In response to TNFα, rat brain pericytes *in vitro* produce IL6 and macrophage inflammatory protein 1 (MIP1), which trigger microglial activation (Matsumoto et al., 2014). Activated microglia disrupt the BBB, which triggers angiogenesis (Dudvarski Stankovic et al., 2016; Shigemoto-Mogami et al., 2018). In the mouse brain, pericytes initially respond to the systemic inflammation within 2 h and secrete CCL2 before the response of astrocytes or microglia (Duan et al., 2018). Given that CCL2 is also known to activate microglia (He M. et al., 2016; Zhang et al., 2017) and microglial process motility dynamics are altered 48 h after systemic infection (Gyoneva et al., 2014), pericytes might modulate microglial process motility and physical dynamics around the vessels in response to infection. Furthermore, pericytes themselves acquire a microglial phenotype after ischemic stroke as mentioned above (Özen et al., 2014; Sakuma et al., 2016). Conversely, pericytes also secrete several anti-inflammatory substances such as IL33 and CX3CL1 (Rustenhoven et al., 2016, 2017; Yang et al., 2016), both of which has shown promote anti-inflammatory microglial phenotype in mouse models (Cardona et al., 2006; Fu et al., 2016).

### Perivascular Macrophages and Pericytes

In the human and mouse brain, perivascular macrophages lie under the basement membrane alongside pericytes (Fabriek et al., 2005; Goldmann et al., 2016). Perivascular macrophages maintain tight junctions between endothelial cells and limit vessel permeability, phagocytose potential pathogens before they enter tissues from the blood and restrict inappropriate inflammation (Zenker et al., 2003). Although pericytes and perivascular macrophages are localized close to each other and possess shared functions including regulation of vascular permeability and phagocytosis, little is known about how pericytes interact with perivascular macrophages in the vascular niche (Lapenna et al., 2018).

## Pathological Roles of Pericytes in Cerebrovascular Diseases and AD

Blood–brain barrier breakdown and microvessel dysfunction has been observed in various CNS disorders such as small vessel disease (SVD), ischemic acute stroke, intracerebral hemorrhage, Alzheimer’s disease (AD), traumatic brain injury (TBI)/chronic traumatic encephalopathy (CTE), multiple sclerosis (MS), amyotrophic lateral sclerosis (ALS), Lewy body diseases (LBD), and epilepsy (Winkler et al., 2013; Coatti et al., 2019; Erdener and Dalkara, 2019; Geranmayeh et al., 2019). In particular, pericyte dysfunction is thought to be a critical factor for aggravating dementing diseases such as vascular cognitive impairment/dementia and AD (Sagare et al., 2013; Montagne et al., 2018; Nikolakopoulou et al., 2019).

Cognitive impairment/dementia related to vascular pathology is classified according to the causative vessel size, that is, ‘small vessel’ disease and ‘large vessel’ disease, although their crosstalk would be essential for the pathogenesis of both disorders (Ihara and Yamamoto, 2016). Cerebral SVD contributes to a wide range of pathological processes, which affect the small vessels including small arteries, arterioles, venules, and capillaries in the brain (Østergaard et al., 2016; Staszewski et al., 2017; Parkes et al., 2018). In contrast, large vessel disease in the brain may result in stroke and hemorrhage, which affect various type of arteries (Nomura et al., 2018). Table 2 provides the roles of CNS pericytes in health and disease focusing on cerebrovascular diseases and AD.

**TABLE 2:** The roles of CNS pericytes in health and disease.

**Pericyte functions**	**Pericyte roles under pathological conditions**			**References**
	**SVD**	**Stroke**	**AD**	
**BBB maintenance**	BBB breakdown and white matter attenuation	BBB breakdown and causes hemorrhagic stroke	BBB breakdown and white matter attenuation	Armulik et al. (2010), Bell et al. (2010), Daneman et al. (2010), Seo et al. (2014), Nikolakopoulou et al. (2019), Stebbins et al. (2019)
**Angiogenesis**	Compensatory angiogenesis	Revascularization and blood vessel stabilization		Liu et al. (2000), Zechariah et al. (2013), Durham et al. (2014), Eilken et al. (2017), Teichert et al. (2017), Berthiaume et al. (2018), Blocki et al. (2018)
**Regulation of CBF (neurovascular coupling)**		Capillary constriction and no-reflow phenomenon after stroke	Capillary constriction and CBF reduction	Yemisci et al. (2009), Bell et al. (2010), Fernández-Klett et al. (2010), Hamilton et al. (2010), Hall et al. (2014), Hill et al. (2015), Kisler et al. (2017b), Cai et al. (2018), Khennouf et al. (2018)
**Clearance of the brain**	Trap toxic substances	Trap toxic substances	Aβ clearance	Armulik et al. (2010), Bell et al. (2010),Sagare et al. (2013), Schultz et al. (2017), Ma et al. (2018)
		Acquire microglial properties		Özen et al. (2014), Sakuma et al. (2016)
**Immunological property**	Release inflammatory substances	Release of pro- and anti-inflammatory substances	Release inflammatory substances	Kovac et al. (2011), Proebstl et al. (2012), Guijarro-Muñoz et al. (2014), Herland et al. (2016), Rustenhoven et al. (2016), Ogura et al. (2017), Duan et al. (2018), Matsumoto et al. (2018), Smyth L.C.D. et al. (2018)
**Stem cell-like property**		Change to microglia-like cellsand stem cells		Özen et al. (2014), Nakagomi et al. (2015a), Sakuma et al. (2016)
**Scar formation**	Astrogliogenesis	Make barrier between infarcted and intact area		Göritz et al. (2011), Makihara et al. (2015), Dias et al. (2018), Hesp et al. (2018), Uemura et al. (2018)

*Aβ, amyloid beta; BBB, blood–brain barrier; CBF, cerebral blood flow.*

### Small Vessel Disease

SVD is characterized by pathological changes in the small vessels with a diameter < 100 μm, with concentric smooth muscle thickening in arterioles, as well as pericyte degeneration, basal membrane thickening, endothelial, and astrocyte end-feet swelling in capillaries (Craggs et al., 2014; Bosetti et al., 2016; Østergaard et al., 2016). The slowly progressive worsening of microcirculatory structure and function results in white matter changes, which can be detected by magnetic resonance imaging (MRI).

Small vessel disease is commonly known to be co-morbid brain pathology in a wide range of neurodegenerative diseases including CTE, MS, LBD, and diverse tauopathies including AD (Erdener and Dalkara, 2019). In sporadic SVD, pericytes play pivotal roles as they reside in the small vessels and contribute to maintenance of the BBB, vascular integrity, inflammation, and angiogenesis. Chronic hypoperfusion in the rodent brain results in degeneration of pericytes and decreased pericyte coverage in brain blood vessels, and increased BBB permeability followed by white matter attenuation (Ueno et al., 2002; Liu Q. et al., 2019). Pericyte-deficient mice also cause circulatory failure in the brain which can trigger white matter functional deficits and neuronal loss (Bell et al., 2010; Montagne et al., 2018; Nikolakopoulou et al., 2019).

A leaky BBB allows for the extravasation of toxic-blood derived products such as fibrinogen, which accumulates around the vasculature as insoluble fibrin (Bell et al., 2010; Montagne et al., 2018; Nikolakopoulou et al., 2019). Fibrinogen/fibrin infiltration results in clustering and activation of macrophages and microglia as well as chemokine- and antigen presentation-mediated recruitment and activation of T cells, causing axonal degeneration (Davalos et al., 2012; Ryu et al., 2015). Pericytes produce numerous pro-inflammatory mediators including reactive oxygen/nitrogen species (ROS/RNS), which induces neurons to undergo stress-induced apoptosis (Rustenhoven et al., 2017). This pro-inflammatory status in the vessels induces leukocyte adhesion and microglial activation (Matsumoto et al., 2018; Erdener and Dalkara, 2019). Under chronic hypoperfusion, bone morphogenetic protein 4 (BMP4) expression is increased by pericytes, which induces astrogliogenesis and aggravates white matter attenuation (Uemura et al., 2018).

Chronic hypoperfusion induces compensatory angiogenesis by increasing the expression of angiogenetic factors such as VEGF, ANGPT1/2, and MMP9 (Jian et al., 2003; Ohtaki et al., 2006; Min-Soo et al., 2018). VEGF and ANGPT1 promote sprouting and proliferation of endothelial cells, and recruitment of pericytes (Shane and Didier, 2011). MMP9 regulates the detachment of pericytes from vessels thereby triggering angiogenesis (Joyce, 2005).

The importance of pericytes in SVD may be emphasized by the fact that one of the most common inherited cerebral SVD, cerebral autosomal dominant arteriopathy with subcortical infarcts and leukoencephalopathy (CADASIL), shows aggregation of mutant Notch3 protein around capillary pericytes as well as arteriolar SMCs (Ihara and Yamamoto, 2016). Pericytes express Notch3 and are first affected by Notch3 aggregation in *Notch3*^*R169C*^ mice, suggesting pericytes might be a main contributor in the pathogenesis of CADASIL (Ghosh et al., 2015). A recent study, however, has shown no change in pericyte coverage in the white matter lesion of CADASIL patients nor *Notch3*^*R169C*^ mice, arguing against the prevailing hypothesis that pericyte loss is the primary driver of white matter lesions (Rajani et al., 2019). The jury is still out on the contribution of pericytes to the white matter damage.

### Cerebral Ischemic Stroke

Residing in the microvessels, pericytes have a great influence on the condition of the brain following acute ischemic stroke caused by thrombosis or embolism affecting larger vessels. The biological roles of pericytes, such as regulation of CBF, BBB maintenance, inflammation and immunological properties, angiogenesis, and scar formation are all involved in the status of the ischemic brain (Gautam and Yao, 2018).

During arterial obstruction, pericytes positioned on the proximal capillaries constrict the vessels and impede capillary blood flow, which lasts even after arterial recanalization, developing a no-reflow phenomenon (Dalkara and Arsava, 2012; Gursoy-Ozdemir et al., 2012; Hall et al., 2014, Hill et al., 2015). The debate about whether these cells are pericytes or SMCs was discussed above.

During stroke, BBB permeability is increased, and sustained ischemia leads to increased BBB disruption (Simpkins et al., 2016). Severe BBB disruption during stroke increases the risk of hemorrhage in patients treated with intravenous tissue-type plasminogen activator (Deguchi et al., 2014). During ischemia, as in SVD, ROS production and MMP9 up-regulation in pericytes contribute to BBB breakdown. An enzymatic source of ROS production, nicotinamide adenine dinucleotide phosphate oxidase 4 (NOX4), is highly up-regulated by pericytes in the peri-infarct region of the mouse brain subjected to middle cerebral artery occlusion (MCAO), and overexpression of NOX4 in pericytes induces BBB breakdown by up-regulating MMP9 (Nishimura et al., 2016). Pericytes also directly release MMP9 during ischemia, which interrupts the tight junctions between endothelial cells and the binding of astrocyte endfeet to the vascular wall (Underly et al., 2017). VEGF up-regulation by pericytes under ischemic conditions has also been reported to disrupt the BBB *in vitro* and *in vivo* (Zheng Gang et al., 2000; Bai et al., 2015), and this triggers further angiogenesis. However, a report has shown that prolonged exposure to VEGF enhances post-ischemic BBB integrity and reduces infarct volume in mice subjected to transient MCAO (Zechariah et al., 2013). Thus, VEGF might have a pluripotent role in BBB integrity according to the dosage and timing of its release.

Pericytes might also play a role in regulating ischemia-induced leukocyte infiltration as pericytes express cell surface adhesion molecules and induce leukocyte transmigration in response to inflammatory mediators (Balabanov et al., 1999; Pieper et al., 2013; Stark et al., 2013). Pericytes express ICAM-1, which guide leukocyte migration through gaps between pericytes by interacting with the integrin ligands on leukocytes (Proebstl et al., 2012).

Aside from these detrimental roles, pericytes play a beneficial role in ischemic stroke via promoting angiogenesis and scar formation. During MCAO-inducing ischemia in the mouse brains, pericytes are recruited from the periphery as well as parenchyma, and are involved in angiogenesis and blood vessel stabilization (Renner et al., 2003; Kokovay et al., 2006). Pericyte migration to the infarcted area forming the core of the scar, which is distinct from the astroglial scar surrounding the core (Fernández-Klett et al., 2013). Consistent with that, *Pdgfrb*^+ ⁣/−^ mice demonstrated decreased fibrosis in the ischemic area and enlarged infarct volume (Makihara et al., 2015). Although glial scar formation is beneficial to prevent toxic substances from spreading, it should be noted that excessive or long-lasting glial scar formation inhibits axonal regeneration and stalls the recovery process (Dias et al., 2018). Furthermore, detachment of pericytes from capillaries allows them to migrate toward ischemic region thereby causing further leakage of the BBB. The same is true of angiogenesis. While angiogenesis increases the blood supply to the peri-infarct area, insufficient angiogenesis results in leaky blood vessels leading to brain hemorrhage (Kuhnert et al., 2010; Cullen et al., 2011).

### Alzheimer’s Disease

AD is the most prevailing dementia among the elderly and is defined pathologically by the presence of Aβ accumulation in brain parenchyma as Aβ plaque and aggregation of hyperphosphorylated tau as neurofibrillary tangles as well as neuritic plaques and neuropil threads. Aβ also accumulate in the vessels as cerebral amyloid angiopathy (CAA). Recently, it has been increasingly recognized that the decreased CBF and white matter attenuation associated with BBB breakdown correlates with the accumulation of AD pathology, and contributes to the onset and progression of dementia (Iturria-Medina et al., 2016; Leijenaar et al., 2017; Park et al., 2019). CBF reduction, BBB breakdown in the hippocampus, and an increase in PDGFRβ level in the CSF occur even in the very early stages of cognitive impairment (Iris et al., 2007; Montagne et al., 2015; Iturria-Medina et al., 2016; Nation et al., 2019) as well as later stages of AD (Miners et al., 2019). In AD patient brains, microvessels are frequently narrowed and irregular in diameter especially in the vicinity of the senile plaques, which is accompanied by decreased capillary bed densities (Kitaguchi et al., 2007). Some vessels in these area are collapsed with lacking endothelial cells, and do not carry blood flow, called string vessels (Hunter et al., 2012). In the mouse brain, infusion of Aβ caused endothelin-1 (ET1) upregulation in cerebral vasculature through receptor for advanced glycation end products (RAGE), which contributes to Aβ-induced CBF reduction (Deane et al., 2003). CBF reduction accompanied by increased vascular RAGE and ET1 is also observed in Tg2576 mice, which is ameliorated by blocking Aβ and RAGE binding (Deane et al., 2003). A recent study has shown that capillaries in the AD brains are constricted by pericytes, which causes a decrease in CBF (Nortley et al., 2019). In rat brains, Aβ oligomer-induced ROS triggers the release of ET1 to stimulate pericytes contraction and CBF reduction (Nortley et al., 2019). AD patients also show a decrease in pericyte coverage with an increase in extravascular immunoglobulin G and fibrin deposition (Sengillo et al., 2013). The apolipoprotein E4 genotype, which is a major genetic risk factor for late-onset AD, leads to pericyte loss and enhances CypA-MMP9 pathway of BBB degradation (Halliday et al., 2016). Pericytes express LRP1 and other Aβ-binding receptors such as the low density lipoprotein receptor (LDLR), RAGE, and CD36 in brains with AD pathology including CAA (Zenaro et al., 2017). Aβ accumulation in pericytes is observed in human AD brains and in the brains of *APP*^*sw/0*^ mice (Ma et al., 2018). At the ultrastructural level of AD brains, pericytes are disorganized and exhibit mitochondrial abnormalities, pinocytotic vesicles, and accumulation of osmophilic material (Farkas and Luiten, 2001; Baloyannis and Baloyannis, 2012).

Loss of BBB integrity caused by pericyte deterioration may induce an influx of immune cells into the brain, driving inflammation, and CBF stagnation and thereby impairing Aβ clearance, all of which aggravate AD pathology (Mazza et al., 2011; Kinney et al., 2018). Indeed, both the depletion of pericytes in the *APP*^*sw/0*^ mice (*APP^*sw/0*^; Pdfgfb^+ ⁣/−^*) (Sagare et al., 2013) and chronic cerebral hypoperfusion in the *APP*^*SwInd*^ Tg mice (Kitaguchi et al., 2009; Yamada et al., 2011) aggravate AD pathology such as increasing Aβ deposition and tau phosphorylation followed by neuronal loss. Notably, a high-fat diet, which leads to vascular related diseases, exacerbates AD pathology accompanied by pericyte dysfunction in the *APP*^*sw/PS1*^ mice (Theriault et al., 2016).

Although Aβ is toxic to the pericytes, pericytes basically take an active part in the clearance of Aβ by phagocytosis and translocation through BBB (Winkler et al., 2014; Alla et al., 2019). Pericytes clear Aβ aggregates via an LRP1/ApoE isoform-specific mechanisms, suggesting a potential therapeutic target for controlling Aβ clearance in AD (Winkler et al., 2014; Ma et al., 2018).

## Therapeutic Strategies Focusing on Pericytes

Since pericytes have multifunctional properties and contribute to the various neurological disorders, pericytes as a therapeutic target, can be approached from various aspects: (1) prevention of BBB dysfunction, (2) promoting angiogenesis and vascular stability, (3) reduction of pericyte constriction under pathological condition, (4) up-regulation of Aβ clearance, (5) control of inflammation, (6) implantation therapy through multipotential stem cell properties, and (7) regulation of proper scar formation.

As the BBB tightly restricts the passage of substances into the CNS, it is challenging to deliver drugs from blood circulation into the brain. Therefore, the delivery methods which enable the drugs to pass through BBB may be beneficial as exemplified by encapsulating drugs in liposomes or nanoparticles (Gaudin et al., 2014; Fullstone et al., 2016; Zhou et al., 2018).

### Prevention of BBB Dysfunction, Promoting Angiogenesis, and Vascular Stability

Loss of pericytes and BBB dysfunction are common in a variety of neurological disorders including cerebrovascular diseases and AD. Thus, promoting the interaction of pericytes and endothelial cells by regulating PDGFB/PDGFRβ, TGFβ, Notch, ANGPT/TEK, and VEGF signaling could be therapeutic by preventing BBB dysfunction and facilitating proper angiogenesis and vascular stability. For instance, increasing PDGF-BB in the endothelial cells and/or PDGFRβ in the pericytes could boost pericyte proliferation and migration to microvessels while increasing TGFβ signaling could promote pericyte proliferation and attachment to the vessels (Winkler et al., 2011b). Indeed, administration of TGFβ showed increased BBB formation under ischemic condition *in vitro* and *in vivo* (Shen et al., 2019). VEGF treatment was also shown to enhance post-ischemic BBB integrity and reduce infarct volume in rodent models of MCAO (Zheng Gang et al., 2000; Zechariah et al., 2013). Recently, regulating RNA for maintaining the BBB has been investigated and overexpression of *cPWWP2A* or silencing *microRNA-579* expression promoted pericyte-endothelial cell crosstalk and microvascular stability (Liu C. et al., 2019). Administration of *microRNA-149-5p* attenuated BBB permeability and improved the outcomes of rat subjected with transient MCAO (Wan et al., 2018).

Reduction of ROS and MMP9 should also prevent pericyte-mediated BBB breakdown. A recent study observed that MMP9 inhibitors reduced pericyte-associated BBB leakage (Underly et al., 2017). A free radical scavenger edaravone has been sown to ameliorate brain damage after ischemia via pericyte-mediated angiogenesis and vessel stability (Deguchi et al., 2014). Further, cilostazol – a phosphodiesterase 3 inhibitor – promoted angiogenesis through pericyte proliferation with inhibition of the MMP9, which maintained vascular integrity in spontaneously hypertensive stroke prone (SHR-SP) rat (Omote et al., 2014) while cilostazol also ameliorated cerebral hemorrhage in mice by protecting the BBB (Takagi et al., 2017), suggesting cilostazol has additional effects of vascular stability aside from antithrombosis.

### Reduction of Capillary Constriction by Pericytes Under Ischemia or Aβ Accumulation

Since the no-reflow phenomenon hampers the tissue recovery after recanalization in the arteries, researchers have tried to find clues to prevent capillary constriction by pericytes after ischemia. These ischemia-induced pericyte contraction have shown to be relieved by suppressing ROS/RNS (Yemisci et al., 2009; Deguchi et al., 2014; Hall et al., 2014), removal of external Ca^2+^ (Hall et al., 2014), or administration of adenosine and sodium nitroprusside (Neuhaus et al., 2017; O’Farrell et al., 2017). Capillary constriction by pericytes and CBF reduction are also observed in AD brains, which may aggravate cognitive decline. Aβ infusion into mouse brain causes RAGE-ET1 mediated CBF reduction, similar to the CBF reduction observed in aged Tg2576 mice (Deane et al., 2003). Furthermore, a RAGE-specific inhibitor recovered CBF and lowered the Aβ burden in *APP*^*sw/0*^ mice (Deane et al., 2012). Aβ oligomers induce pericyte constriction by ROS mediated ET1 release (Nortley et al., 2019). This Aβ-evoked constriction was reversed by applying the vasodilator C-type natriuretic peptide and could be halted by blocking NOX4 or ET1 receptors, suggesting potential therapeutic target for CBF reduction in AD.

### Up-Regulation of Aβ Clearance

Because pericytes take up Aβ, then degrade or excrete it into the circulation, boosting pericyte function could be therapeutic target for Aβ clearance in AD. Consistent with that, pericyte loss in the *APP*^*sw/0*^ mice showed increased Aβ accumulation and tau phosphorylation (Sagare et al., 2013). Pericytes internalize and clear aggregated Aβ by LRP1-dependent ApoE isoform-specific mechanism (Ma et al., 2018), highlighting up-regulation of LRP1 as a therapeutic target for Aβ clearance.

### Control of Inflammation

Neuroinflammation is present in almost all neurological diseases. Pericytes release both pro-inflammatory and anti-inflammatory mediators and regulate recruitment of immune cells from the blood to the brain parenchyma. Although inflammation may have some positive aspects such as immunoprotection against pathogens, clearance of toxic substances, and support of angiogenesis, excessive inflammation causes BBB leakage, tissue damages and neuronal loss.

Targeting ROS-mediated inflammation is a feasible therapeutic target in terms of suppressing release of pro-inflammatory cytokines by pericytes with preventing pericyte loss and BBB dysfunction. A free radical scavenger edaravone has been sown to ameliorate brain damage after ischemia (Deguchi et al., 2014). Targeting receptor or downstream signaling stimulated by pericyte-secreted cytokines may also be potential therapeutic target. Blocking pericyte-derived BMP4 by its receptor antagonist noggin treatment was shown to suppress astrogliogenesis and alleviate white matter damage resulting from chronic cerebral hypoperfusion (Uemura et al., 2018). Targeting transcription factors that regulate immune functions following inflammatory insults might be another option to suppress the detrimental effects of inflammation.

### Implantation Therapy, Application of Multipotential Stem Cell Properties

Implantation of mesenchymal stem cell-derived pericytes in mice that model AD plaque pathology reduces the Aβ burden, demonstrating the possibility of cell-based therapy for AD treatment (Tachibana et al., 2018). Easy accessibility of pericytes for autologous transplantation highlights their capabilities for future therapeutic studies (Geranmayeh et al., 2019). Since pericyte-like cells derived from induced pluripotent stem cells (iPSC) acquire BBB properties and are incorporated with iPSC-derived endothelial cells, astrocytes and neurons (Faal et al., 2019; Stebbins et al., 2019), iPSC-derived pericytes might also be promising for implantation therapy of AD and other neurological disorders. Pericytes themselves have been shown to acquire stem cell-like and microglial properties after ischemia (Özen et al., 2014; Nakagomi et al., 2015a), which offers another potential therapeutic target for recovery form CNS diseases.

### Regulation of Proper Scar Formation

As discussed above, scar formation in the ischemic brain and brain/spinal cord injury has pluripotent effects on diverse CNS conditions. While the scar formation by pericytes and glia play fundamental roles in promoting angiogenesis and tissue remodeling (Hesp et al., 2018), reducing pericyte-derived scar formation has been reported to promote axonal regeneration and recovery from spinal cord injury (Dias et al., 2018). Administration of periostin-neutralizing antibody, which suppresses pericyte-induced scar formation, ameliorates functional recovery after spinal cord injury (Yokota et al., 2017), but further studies are needed to demonstrate the therapeutic potential of these strategies including the appropriate timing and degree of intervention.

## Conclusion

All CNS cells and tissues need a blood supply coming from outside the brain. Located at the interface between CNS tissue and blood circulation and having multi-functional properties, pericytes play a variety of fundamental roles in the healthy CNS. As a result, pericytes offer many opportunities for therapeutic intervention in a broad range of neurological disorders, including cerebrovascular disorders and AD. Vascular cognitive impairment/dementia and AD account for more than 3/4 of dementing diseases, and vascular pathology is often observed in a various neurodegenerative disease, especially in AD, where pericytes are thought to contribute. With increasing knowledge about the molecular mechanisms operating in pericytes and their crosstalk with neighboring cells, the targeting pericytes as a therapeutic strategy has become increasingly important and research on this topic is likely to accelerate more in the future.

## Author Contributions

MU conceptualized the study, designed and drafted the manuscript and figures, and handled the funding. TM supervised and critically revised the manuscript for important intellectual content. MI, VL, and JT handled the funding, supervising, and making critical revision of the manuscript for important intellectual content.

## Conflict of Interest

The authors declare that the research was conducted in the absence of any commercial or financial relationships that could be construed as a potential conflict of interest.
